# Mental health and wellbeing of border security personnel: scoping review

**DOI:** 10.1093/occmed/kqac108

**Published:** 2022-10-31

**Authors:** S K Brooks, N Greenberg

**Affiliations:** Department of Psychological Medicine, King’s College London, Weston Education Centre, London SE5 9RJ, UK; Department of Psychological Medicine, King’s College London, Weston Education Centre, London SE5 9RJ, UK

## Abstract

**Background:**

Whilst the wellbeing of law enforcement personnel has been widely researched, border security personnel as a discrete group appear to be far less discussed, despite their roles frequently exposing them to potentially traumatic and challenging events such as contact with criminals or witnessing personal tragedies due to trafficking or smuggling.

**Aims:**

This scoping review aimed to explore existing literature to better understand the mental health of border security personnel and the factors affecting their wellbeing.

**Methods:**

Four electronic databases and grey literature were systematically searched for studies relevant to the review’s aims. Following the extraction of relevant data from each study, thematic analysis was used to synthesize findings.

**Results:**

Thirteen studies included relevant data and were included in the review, identifying stressors including poor management; fatigue; negative public attitudes; inadequate staffing levels, resources, and training; poor opportunities for promotion; low pay; work overload; dangerous working environments; and work-related moral dilemmas.

**Conclusions:**

The review found that there has been little academic attention paid to border security personnel as a specific branch of law enforcement. Many of the stressors identified in this review are those also reported by law enforcement generally, although negative attitudes from the public and exposure to moral dilemmas appear to be more relevant for border security staff. Directly addressing work-related stress (e.g. by fostering a supportive organizational culture, addressing mental health stigma, and encouraging help-seeking) may be useful in enhancing the resilience of border security personnel.

## Teaser

This scoping review revealed that there is little literature focusing specifically on the mental health of border security personnel despite this occupational group facing unique stressors. The review identified various occupational stressors faced by border security staff; moral injury and negative public attitudes appeared to be prevalent and worthy of more research. Fostering supportive organizational cultures may enhance the resilience of border security personnel.

**Key Learning Points**

**What is already known about this subject:**

- Law enforcement personnel frequently experience mental ill-health such as post-traumatic stress disorder and depression.
- Law enforcement staff’s mental health appears to be associated with many occupational and organisational factors, including high demands, workload, work-family conflict, organizational injustice, low autonomy, low reward, inadequate training, inadequate resources, poor support, and traumatic exposure.
- Border security personnel are a distinct branch of law enforcement who have rarely been studied in isolation from other law enforcement personnel.

**What this study adds:**

- There is little research examining the prevalence of stress or mental ill-health in border security personnel, although some evidence suggests their mental health may be poorer than the general population and other law enforcement branches.
- Key stressors appear to be similar to those reported by law enforcement generally, although negative perceptions from the public appear to be a prominent stressor for border security personnel despite rarely being discussed in law enforcement literature.
- There is little research on help-seeking in border security personnel or interventions that may help improve their wellbeing.

**What impact this may have on practice or policy:**

- More research specifically focusing on border security personnel should be carried out, to better understand the similarities and differences between this sub-group and law enforcement more generally in terms of their wellbeing and risk factors for poor mental health.
- The relationship between staff wellbeing and the perceived public image of the organization may be an important area to consider.
- Encouraging a supportive organizational culture, reducing mental health stigma in the workplace, and encouraging help-seeking may improve mental health outcomes for border security personnel; increasing opportunities for career development, improving the organization’s public image, reducing individual workloads, and improving the resources and training available to staff could help foster resilience.

## Introduction

Border security personnel are a group of law enforcement personnel responsible for protecting borders and regulating the movement of goods and individuals across land, air and maritime borders. They perform a wide range of duties and are frequently exposed to trauma, tragedy, and crime—for example, facing direct threats from criminals and frustrated passengers, and having to contend with refugees (including children) who risk drowning trying to enter the country in boats, as well as refugees who risk suffocation in lorries. The role of a border security officer is characterized by the need to remain calm in stressful situations [1] and be constantly alert and ready to make rapid, reactive decisions [2] which may be morally challenging and could potentially lead to moral injury [3]. Staff regularly meet a diverse range of cultural groups all of whom are engaged in travel which often may cause the individuals who are travelling to be stressed, tired or at times desperate; this can lead to challenging or stressful interactions with border security personnel which can in turn create stress for border security personnel.

Law enforcement personnel have been frequently identified as an occupational group at risk for mental health problems [4] including post-traumatic stress disorder (PTSD) [5], depression [6] and alcohol misuse [7], given the potentially traumatic nature of their work, such as close contact with criminals and witnessing dangerous or tragic events. Whilst a wealth of literature examines wellbeing and mental ill-health in law enforcement personnel, research specifically focusing on border security personnel is scarce [8].

Literature reviews have established various factors associated with the mental health of law enforcement personnel. Many are work-related, including high demands, heavy workload, work-family conflict, perceived organizational injustice, lack of autonomy, lack of reward, inadequate training and inadequate equipment [4, 9–11]. Other key predictors of poor wellbeing appear to be a lack of organizational support [9, 10], exposure to traumatic events [11] and high cumulative critical incident exposure [4, 10]. Additionally, officers are often reluctant to seek mental health support due to negative perceptions of mental health support and concerns about job confidentiality and potential consequences on their careers [12].

It is possible that border security personnel have been included in existing research, but most studies do not differentiate between their data and that of other law enforcement staff [8]. Although it is possible that the mental health of border security personnel is comparable to law enforcement staff in general, they are a distinct entities. For instance, Border Patrol personnel in the United States perceive that they have low pay and poor working conditions compared to other law enforcement roles [13]. It is therefore possible that they may differ from other agencies in terms of who applies for these roles, the prevalence of mental ill-health and its predictors. This review fills a gap in the literature by collating, for the first time, research focusing specifically on the mental health and wellbeing of border security personnel.

## Methods

As the literature on border security personnel has never been synthesized before and it was unclear how many studies we would find, a scoping review methodology was selected as such reviews are typically broader in scope and inclusion criteria than systematic reviews. This review followed Arksey and O’Malley’s [14] scoping review framework; a protocol was developed but not pre-registered. As we were interested in finding any literature relating to the broad area of mental health and wellbeing in border security staff, a broad research question was developed by the authors: *What is known about the mental health and wellbeing of border security personnel?*

The first author searched PsycInfo, Medline, Embase and Global Health databases using a combination of search terms relating to border security and wellbeing; the full search strategy is available in Supplementary Appendix 1. The search strategies were devised by the first author and refined through discussion with the second author. To supplement these searches, combinations of relevant keywords were also searched using OpenGrey and Google Scholar and reference lists of included literature were hand-searched. Searches were carried out in July 2021 and updated in April 2022. Due to the paucity of research in this area, there were no limits in terms of year of publication, type of publication or language. The only inclusion criteria were: literature needed to present primary data on the mental health or wellbeing of border security personnel (in order to be relevant to the review) and studies must have a sample size greater than *n* = 1 (to allow for generalizations from the data).

All citations were imported into EndNote reference management software, where one author (SKB) reviewed titles, abstracts and full texts for relevance. Any clearly not meeting the inclusion criteria were excluded and any uncertainties about including a study were discussed with the second author. Relevant data from included studies were extracted onto a pre-designed spreadsheet with the following headings: authors, year of publication, country, type of publication, number of participants, job role of participants, socio-demographic characteristics of participants, design, variables measured, instruments used, key results and limitations. Thematic analysis [15] was used to synthesize the data, identifying key concepts or ‘themes’ in the included studies and recognizing similar concepts across studies (for example, all data relating to rates of mental health problems in the study population was coded as ‘prevalence of mental health problems’ and all data relating to factors related to mental health/wellbeing was coded as ‘factors associated with mental health/wellbeing’). Data saturation was achieved when all relevant data extracted from included studies had been coded and no new themes emerged. Both authors reviewed the resultant data and discussed emergent themes and any anomalies. Each of the themes identified is summarized below with narrative descriptions of the evidence found within each theme.

No ethical approval was required for this study as no primary data was collected.

## Results

A total of 96 citations were found via database searching: 71 were excluded based on title, 16 based on abstract and one based on full text. One study with a potentially relevant abstract could not be fully screened due to the article being unavailable online. Another six studies were located via grey literature searching or hand-searching of reference lists, resulting in a total of thirteen studies for inclusion.

Ten included studies were journal articles [2, 8, 16–23], two were doctoral theses [24, 25] and one was a Master’s dissertation [26]. Sample sizes ranged from 5 to 722. An overview of the characteristics of the included studies is presented in Supplementary Appendix 2: Table S1 while details of each study are presented in Supplementary Appendix 3: Table S2.

The first theme (‘prevalence of mental health problems’) revealed that very few studies assessed the prevalence of mental health problems in border security personnel. Two studies reported that border security police had a high prevalence of experiencing potentially traumatic events [8, 16], but none directly assessed post-traumatic stress symptoms. There was some evidence that border security personnel may have poorer mental health than both police officers generally [8] and the general population [8, 17]. The impact of stress on health was rarely considered, with limited data within the second theme (‘impact of stress on health’) suggesting stress may be associated with poor physical health [8], nicotine dependence [21] and emotional problems [8, 24].

The third theme identified was ‘factors associated with mental health/wellbeing’. Within this theme, there was some evidence that poorer wellbeing was associated with work-related factors such as high workload, lack of control at work and poor perceived rewards [2]. Limited evidence suggested that stress may be higher in older staff [21], less educated staff [21], staff with low emotional intelligence [17] and staff who smoked cigarettes [21] and there were mixed findings as to the association between marital status and occupational stress [8, 21].

The fourth theme (‘causes of stress’) found several stressors reported by border security personnel. These included inadequate sleep and rest [17, 20] and fatigue [20, 26], perhaps related to work overload and irregular work hours [8]. Relationships with managers appeared to be important, with unsupportive management and poor leadership reported to cause stress [8, 17, 20, 24, 26]. Other work-related stressors included leave-related issues [17]; inadequate staffing levels [24, 26] and resources [8, 17, 24]; low salary [8, 17]; disadvantageous conditions as compared to police in general [8]; lack of colleague support [8] and the dangerous aspects of the job such as difficult physical working conditions [8, 24], fear of injury [8], and working with hostile populations [8, 24]. Negative public perceptions were also stressful, including fear of assaults [8, 24] and being shamed for the nature of their work [20]. A list of reported stressors is presented in Supplementary Appendix 3: Table S2.

Participants reported various ‘moral challenges’ such as performing tasks they did not agree with [8] and the dilemma between fulfilling their job role and the fundamental human feelings of empathy and compassion towards immigrants and the tension between feeling pride in their work and feeling ashamed of the morally questionable aspects [20]. Whilst a quantitative study [2] found no significant relationship between perceived occupational stress and conflict between employees’ values and organizational values, a qualitative study [20] found that moral tensions could involve feelings of guilt, helplessness, and not doing enough to help people.

The theme ‘attitudes towards psychological help’ revealed that over a third (of *n* = 168) of participants would feel uneasy about their colleagues knowing about them seeking psychological support [16]; peer-provided psychological support was described as enhancing empathy, but peer support providers risked developing their own trauma symptoms [25].

The final theme, ‘coping’, revealed that the coping strategies most often employed by border security personnel included trying to remain positive and taking with colleagues about problems [8], while those who used disengagement coping (strategies aimed at diverting from the stressor and associated emotions, rather than facing them) were more likely to experience stress [26]. There was some evidence that using yoga may help personnel cope with job stress [22, 23].

## Discussion

This scoping review of literature on the mental health and wellbeing of personnel working in border security described thirteen articles. We aimed to explore how much literature exists looking specifically at border security staff and synthesize the existing literature to provide an understanding of the prevalence of mental ill-health in this population and factors associated with their wellbeing. There was little research on the prevalence of stress or mental ill-health in border personnel although two studies suggested their mental health might be poorer than the general population. Whilst participants in many studies reported being exposed to trauma, there has been little investigation into the impact of traumatic exposure on border security personnel. One study suggested high workload, poor control at work and poor rewards were associated with stress. When asked to describe their main causes of stress, participants reported several different stressors relating to their roles, management and organizational culture. There was very little literature relating to help-seeking or interventions. One study found evidence that border security personnel may be reluctant to seek psychological help, whilst another found that personnel who provide peer support to colleagues may be at risk of vicarious traumatization. Participants described various coping strategies such as exercise and positive thinking, and one study (described in two papers) suggested yoga might help improve sleep and reduce anxiety.

This review fills a gap in the literature by including only studies which presented data specifically on border security personnel. However, the review has limitations which means results must be interpreted with caution. First, it is difficult to synthesize literature on border security personnel from different countries because the roles and responsibilities of border security differ between countries; the levels of threat and danger faced by border staff as well as the conditions and terrain they work in are likely to differ by country. Results from studies focusing on one country may therefore not be generalizable to others, although many similarities were found in this review. Second, only a small number of articles were found, again suggesting the results may not be generalizable to the wider population of border security personnel. The finding of only a small number of articles appears to reflect a gap in the literature in terms of studying the mental health and wellbeing of border security staff. In addition, whilst the inclusion of grey literature provided us with several important studies, it must be noted that these would not have been subjected to the same rigorous peer-review process as published articles. Finally, a limitation of the review process itself is that only one author carried out the screening process; ideally, all citations would have been double-screened in order to ensure that no relevant studies were excluded.

The key stressors for border security personnel appear to be poor management; fatigue and lack of adequate rest; negative attitudes from the public; inadequate staffing levels; lack of resources; inadequate training; lack of opportunities for promotion; low pay; work overload; working in dangerous environments and experiencing moral dilemmas. Many of these stressors are those also reported by law enforcement generally [3, 4, 9–11]. However, negative attitudes from the public have rarely been discussed in previous reviews of law enforcement mental health. It is unclear whether negative public perceptions are a bigger issue for border security staff than law enforcement generally, or whether public image has simply been overlooked as a potential stressor in other law enforcement studies. There was some evidence that border security personnel were reluctant to seek help for mental health problems due to stigma and fear of repercussions; again, this reflects law enforcement generally [12].

This review’s findings have several implications. First, and perhaps most importantly, more research focusing specifically on border security personnel is needed because currently, very little research on this topic exists. Additional research investigating factors affecting the mental health of border security personnel would allow a greater understanding of the stressors this group face and how these stressors might impact their psychological health which in turn could lead to a better understanding of how best to support this occupational group and how to prevent mental health problems. Further research on the relationship between staff wellbeing and the perceived public image of their organization—for both border security personnel and law enforcement more generally—would also be beneficial, as this review provides some data to suggest that public image might be an important factor affecting staff wellbeing but given the paucity of research examining this, replication of this finding is needed. In addition, given we found some evidence that moral challenges were a stressor for border security personnel, further research exploring potential moral injuries in this population would also be useful. The challenges of having to uphold the law and regulations whilst dealing with human tragedy and suffering amongst those who seek to enter countries illegally could potentially result in moral injury and the topic is deserving of further exploration.

Despite the limited amount of data relating specifically to border security, several potential recommendations can be made. First, it is notable that several reported stressors related to managers (such as lack of managerial support, lack of clarity about expectations, and high pressure from superiors); many other work-related stressors also related to issues that managers may have some control over (such as workload, inadequate leave, limited breaks and poor camaraderie within teams). Therefore, managers could positively affect the levels of stress in their staff by addressing these issues as much as possible. A systematic review that explored the impact of leader behaviours on employee wellbeing found that managerial support, empowerment, and positive relationships between managers and employees had a positive impact on employees’ wellbeing and ability to cope with stress [27]. In addition, an overview of the management of traumatic stress in organizations [28] suggests that, as many of the risk factors for poor wellbeing can be influenced by managers, the relationship between leaders and managers is key and managers should understand how to support their staff appropriately. Therefore, training for managers on how to support employees may be useful.

Interventions that directly address work-related stress, for example by providing training in resilience and coping strategies, may help border security personnel manage their stress: for example, a systematic review of interventions for anxiety, PTSD, and fatigue in law enforcement [29] reported strong evidence to support the use of psychosocial interventions, including those addressing resilience strategies and coping processes. Additionally, current National Institute for Health and Care Excellence guidelines [30] recommend providing staff with resilience and coping strategies such as mindfulness training and stress management training. Taking steps to improve the organizational culture (such as fostering a more supportive environment and reducing stigma around mental health) could facilitate help-seeking when needed [12]. Other recommendations to potentially improve staff wellbeing include increasing opportunities for self-development and career advancement; taking steps to improve the organization’s public image; improving resources and training available to staff; and ensuring that supervisors feel confident to speak to staff about traumatic events or that formal peer support processes used by other trauma-exposed organizations are utilised [28]. Indeed, many of these suggestions have also been reported by border security personnel themselves to be desirable [8, 17].

Improving organizational culture, public image and opportunities for career advancement may also make the role of border security officer more attractive and encourage more people to pursue this career. This could result in increased staff numbers; in other words, incentives for hiring and retaining staff could help to ensure sufficient manpower, allowing officers to share their workload and have adequate sleep and rest and reduce work overload [13].

Overall, this study found that the risk factors for poor mental health in border security personnel appear to be similar to those for law enforcement generally. Therefore, interventions and workplace policies which have been found to improve mental health in law enforcement may be similarly effective in improving the wellbeing of border security officers, although their effectiveness with this sub-group would need to be tested and evaluated.

## Supplementary Material

kqac108_suppl_Supplementary_Material

## References

[1] Chia YSD , Heng WC, Goh LY, Ang CHJ. Job competencies of border security officers in Singapore. J Police Crim Psychol 2021;36:132–144.

[2] Chudzicka-Czupala A , Stasila-Sieradzka M, Dobrowolska M, Kulakowska A. Assessment of worklife areas and stress intensity among border guard officers. Med Pr 2018;69:199–210. 29372912 10.13075/mp.5893.00664

[3] Lentz LM , Smith-MacDonald L, Malloy D, Carleton RN, Bremault-Phillips S. Compromised conscience: a scoping review of moral injury among firefighters, paramedics and police officers. Front Psychol 2021;12:639781. 33868111 10.3389/fpsyg.2021.639781 PMC8044342

[4] Syed S , Ashwick R, Schlosser M, Jones R, Rowe S, Billings J. Global prevalence and risk factors for mental health problems in police personnel: a systematic review and meta-analysis. Occup Environ Med 2020;77:737–747. 32439827 10.1136/oemed-2020-106498

[5] Huddleston L , Stephens C, Paton D. An evaluation of traumatic and organizational experiences on the psychological health of New Zealand police recruits. Work 2007;28:199–207. 17429146

[6] Wickramasinghe ND , Wijesinghe PR, Dharmaratne SD, Agampodi SB. The prevalence and associated factors of depression in policing: a cross sectional study in Sri Lanka. SpringerPlus 2016;5:1776. 27795918 10.1186/s40064-016-3474-9 PMC5061667

[7] Violanti JM , Slaven JE, Charles LE, Burchfiel CM, Andrew ME, Homish GG. Police and alcohol use: a descriptive analysis and associations with stress outcomes. Am J Crim Law 2011;36:344–356.

[8] Malach-Pines A , Keinan G. Stress and burnout in Israeli border police. Int J Stress Manag 2006;13:519–540.

[9] Finney C , Stergiopoulos E, Hensel J, Bonato S, Dewa CS. Organizational stressors associated with job stress and burnout in correctional officers: a systematic review. BMC Public Health 2013;13:82. 23356379 10.1186/1471-2458-13-82 PMC3564928

[10] Sherwood L , Hegarty S, Vallieres F et al. Identifying the key risk factors for adverse psychological outcomes among police officers: a systematic literature review. J Trauma Stress 2019;32:688–700. 31553502 10.1002/jts.22431

[11] Wagner SL , White N, Fyfe T et al. Systematic review of posttraumatic stress disorder in police officers following routine work-related critical incident exposure. Am J Ind Med 2020;63:600–615. 32419181 10.1002/ajim.23120

[12] Richards NK , Suarez EB, Arocha JF. Law enforcement officers’ barriers to seeking mental health services: a scoping review. J Police Crim Psychol 2021;36:1–9.

[13] Nuñez-Neto B. Border Security: The Role of the US Border Patrol [Internet]. Washington DC (US): Congressional Research Service; [updated 2008; cited 2022 Apr 6]. Available from: http://digitalcommons.ilr.cornell.edu/key_workplace/571/

[14] Arksey H , O’Malley L. Scoping studies: towards a methodological framework. Int J Soc Res Methodol 2005;8:19–32.

[15] Braun V , Clarke V. Using thematic analysis in psychology. Qual Res Psychol 2006;3:77–101.

[16] Argustaitė-Zailskienė G , Digrytė L, Žemaitienė, N. Lithuanian State Border Guard Service officers’ traumatic experiences and their psychological counselling needs and attitudes. Health Psychol Rep 2019;7:183–190.

[17] Chhabra M , Chhabra B. Emotional intelligence and occupational stress: a study of Indian Border Security Force personnel. Police Pract Res 2013;14:355–370.

[18] Lee V , Ang J, Neo A, Goh LY, Liew N. Operational vigilance in border security: the Singapore experience. J Police Crim Psychol 2019;34:330–339.

[19] Rivera KD. (2014). Emotional taint: making sense of emotional dirty work at the U.S. Border Patrol. Manag Commun Q 2014;29:198–228.

[20] Rivera KD , Tracy SJ. Embodying emotional dirty work: a messy text of patrolling the border. Qual Red Organ Manag 2014;9:201–222.

[21] Sandhu KS , Arora V, Gupta N, Gupta P, Raja M, Mehta N. Association of occupational stress factors on nicotine dependence among patients visiting dental care unit of Indo-Tibetian border police force station in India. Rocz Panstw Zakl Hig 2016;67:69–74. 26953584

[22] Telles S , Gupta RK, Verma S, Kala N, Balkrishna A. Changes in vigilance, self rated sleep and state anxiety in military personnel in India following yoga. BMC Res Notes 2018;11:518. 30055628 10.1186/s13104-018-3624-y PMC6064066

[23] Telles S , Kala N, Gupta RK et al. Effect of yoga on vigilance, self rated sleep and state anxiety in Border Security Force personnel in India. Work 2019;63:243–251. 31156205 10.3233/WOR-192925

[24] Hamburger H. Southwest Border Patrol Agent Perceptions of Job-Related Threats and Dangers [doctoral dissertation]. Minnesota (USA): Walden University; 2018 [cited 2021 Jul 14]. Available from: https://scholarworks.waldenu.edu/dissertations/5874/

[25] Lennick LC. Because It Needs To Be Done: The Psychological Consequences for Members of the U.S. Border Patrol’s Peer Support Program [doctoral dissertation]. California (USA): Saybrook University; 2018 [cited 2022 Apr 5]. Available from: https://www.proquest.com/docview/2023816958/4A904C842924ADAPQ/1?accountid=11862

[26] Prasad LR. Behind the Front Line: Stressors and Coping in Border Services Officers [Master’s dissertation]. Canada: University of British Columbia; 2006 [cited 2021 Jul 14]. Available from: https://open.library.ubc.ca/cIRcle/collections/ubctheses/24/items/1.0073353

[27] Skakon J , Nielsen K, Borg V, Guzman J. Are leaders’ well-being, behaviours and style associated with the affective well-being of their employees? A systematic review of three decades of research. Work Stress 2010;24:107–139.

[28] Brooks SK , Rubin GJ, Greenberg N. Traumatic stress within disaster-exposed occupations: overview of the literature and suggestions for the management of traumatic stress in the workplace. Br Med Bull 2019;129:25–34. 30544131 10.1093/bmb/ldy040

[29] Lees T , Elliott JL, Gunning S, Newton PJ, Rai T, Lal S. A systematic review of the current evidence regarding interventions for anxiety, PTSD, sleepiness and fatigue in the law enforcement workplace. Ind Health 2019;57:655–667. 30760652 10.2486/indhealth.2018-0088 PMC6885597

[30] National Institute for Health and Care Excellence. Mental Wellbeing at Work: NICE Guideline. [Internet]. 2022 [cited 2022 Sep 12]. Available from: https://www.nice.org.uk/guidance/ng212/resources/mental-wellbeing-at-work-pdf-66143771841733

