# Supplementary material for: Mental health and wellbeing of border security personnel: scoping review
Source: Occup Med (Lond). 2022 Oct 31;72(9):636–40. doi: 10.1093/occmed/kqac108 (PMC9805299; doi:10.1093/occmed/kqac108)
Supplement: kqac108_suppl_Supplementary_Material [file kqac108_suppl_supplementary_material.docx]

**Supplementary material**

**Appendix 1. Sample search strategy**

Full search strategy for PsycInfo (performed July 2021, updated April 2022):

1. border guard*

2. border patrol*

3. border force*

4. border security

5. border police*

6. border control*

7. border troop*

8. frontier guard*

9. frontier police*

10. 1 OR 2 OR 3 OR 4 OR 5 OR 6 OR 7 OR 8 OR 9

11. mental health

12. wellbeing

13. well-being

14. stress*

15. distress

16. ptsd

17. depression

18. anxiety

19. alcohol misuse

20. alcoholism

21. hazardous drinking

22. problematic drinking

23. resilien*

24. 11 OR 12 OR 13 OR 14 OR 15 OR 16 OR 17 OR 18 OR 19 OR 20 OR 21 OR 22 OR 23

25. 10 AND 24

Grey literature was hand-searched using the following individual terms: border security AND mental health, border force AND mental health, border patrol AND mental health, border guard AND mental health, border police AND mental health

**Appendix 2. Table S1: Overview of included studies**

| **Characteristics of studies** | **Results** |
| --- | --- |
| Country of study | United States (n=4)  India (n=4)  Canada (n=1)  Israel (n=1)  Lithuania (n=1)  Poland (n=1)  Singapore (n=1) |
| Design of study | Cross-sectional quantitative (n=5)  Qualitative (n=3)  Ethnographic (n=2)  Mixed-methods (n=1)  Longitudinal (n=1)  Comparative controlled trial (n=1) |

**Appendix 3. Table S2: Characteristics of literature on border security personnel**

| **Authors (year)** | **Country** | **Publication type** | **Participants** | **Participant demographics** | **Design** |
| --- | --- | --- | --- | --- | --- |
| Argustaite-Zailskiene et al. (2019) [16] | Lithuania | Journal article | 168 Lithuanian State Border Guard Service officers | 70% male;  Mean age 32;  Mean length of service 10 years | Cross-sectional quantitative study assessing traumatic exposure and attitudes towards psychological counselling |
| Chhabra & Chhabra (2012) [17] | India | Journal article | 161 Border Security Force personnel | 100% male;  Mean age not reported but 19% aged 20-20, 54% aged 30-45, 27% aged 45+  Mean length of service not reported but 26% less than 10 years, 39% 10-20 years, 35% 20+ years | Cross-sectional study assessing stressful working conditions; open-ended questions to assess participants’ views on how stress could be reduced; occupational stress; and emotional intelligence |
| Chudzicka-Czupala et al. (2018) [2] | Poland | Journal article | 128 Polish Border Guard personnel | 61% male;  Mean age 38;  Mean length of service 16 years | Cross-sectional quantitative study assessing perceived stress and six aspects of the work environment (workload, sense of control, rewards, social support within teams, organisational justice and conflict between employee values and those promoted by the organisation) |
| Hamburger (2018) [24] | USA | Doctoral thesis | 11 former US Border Patrol agents | 82% male;  Mean age not reported, range 30-45;  Length of service not reported | Qualitative study using semi-structured interviews about perceived threats and dangers of being a border patrol agent |
| Lee et al. (2019) [18] | Singapore | Journal article | 12 veteran Border Security officers involved in a variety of border security work including land, air and sea checkpoints and operational planning | 75% male;  Mean age 48;  Mean length of service 23 years | Qualitative study using semi-structured interviews about perceptions of operational vigilance and perceived influences on operational vigilance |
| Lennick (2018) [25] | USA | Doctoral thesis | 5 members of the US Border Patrol’s peer support programme | 60% male;  Age not reported;  Length of service not reported | Qualitative study using interviews to explore the psychological consequences of being a peer support member for Border Patrol |
| Malach-Pines & Keinan (2006) [8] | Israel | Journal article | 497 border police completed questionnaire; 18% of these also took part in an interview | 96% male;  Mean age 27;  Mean length of service 7 years | Mixed-methods study assessing perceived stressors stressors; a single question to assess general stress level; burnout; stress outcomes; coping; structured interview about causes of burnout, significant aspects of work, training for handling stress, and traumatic experiences |
| Prasad (2006) [26] | Canada | Master’s dissertation | 81 Border Services officers working at land border crossings in Western Canada | 57% male;  Mean age not reported, range 20-40;  Mean length of service not reported, but 79% had more than 3 years of service | Cross-sectional quantitative study assessing organisational stress, occupational stress and coping |
| Rivera (2014) [19] | USA | Journal article | US Border Patrol agents; overall ‘n’ not reported, 25 took part in interviews | Not reported | Two-year ethnographic study involving 165 hours of participant observation, 40 hours of interviews and 7 hours of recorded ‘in situ’ conversation; the interviews focused on agent interactions with the public |
| Rivera & Tracy (2014) [20] | USA | Journal article | US Border Patrol agents; ‘n’ not clear | Not reported | Two-year ethnographic study, involving 165 research hours of which approximately 40 were formal interviews with employees; remaining hours consisted of participant observation and shadowing |
| Sandhu et al. (2016) [21] | India | Journal article | 200 Border Police Force personnel | Gender not reported;  Mean age not reported; 11% aged 18-24, 47% aged 25-34, 18% aged 35-44, 18% aged 45-54, 6% aged 55 and over;  Length of service not reported | Cross-sectional quantitative study assessing nicotine dependence and effort, reward and overcommitment |
| Telles et al. (2018) [22] | India | Journal article | 722 Border Security Force personnel | 100% male;  Mean age 31;  Mean length of service 9 years | Longitudinal study assessing the impact of yoga on vigilance, sleep and state anxiety; intervention comprised 240 minutes of yoga (including prayer, yoga postures, breathing techniques and guided relaxation) per day for nine days; vigilance and sustained attention, state anxiety and sleep quality were assessed |
| Telles et al. (2019) [23] | India | Journal article | 112 Border Security Force personnel (vs. 112 private security firm personnel) | 100% male;  Mean age 30 for Border Security Force personnel, 29 for private security personnel;  Length of service not reported | Comparative controlled trial to examine the effects of yoga on vigilance, sleep and state anxiety; intervention as described above |

**Appendix 4. Table S3: Themes and key results**

| **Theme** | **Sub-theme** | **Key findings** |
| --- | --- | --- |
| Prevalence of mental health problems | Suicide  Stress and burnout  Trauma | Indian Border Security Force personnel have a suicide rate substantially higher than that of the general Indian population [17]  In a comparison of Israeli border police with both police officers generally and the general Israeli population, stress and burnout were significantly higher in border police; In this study of 497 personnel, over half reported high or very high levels of stress [8]; however, participants also reported high levels of job satisfaction due to feeling their work was important [8]  Two studies explored the prevalence of having experienced potentially traumatic events, finding that almost three-quarters of n=497 [8] and almost two-thirds of n=168 [16] had been exposed, either directly or indirectly, to trauma at work; however, no studies directly measured post-traumatic stress symptoms |
| Impact of stress on health | N/A | Stress often resulted in physical and emotional problems such as pain, nervousness, and anger [8]  There was an association between occupational stress and nicotine dependence [21]  A qualitative study with a small sample size (n=11) found that participants reported a substantial emotional toll of their work; low morale; and poor quality of life [24] |
| Factors associated with mental health/wellbeing | Workload  Control  Reward  Support  Organisational justice  Socio-demographic and work characteristics | High workload was associated with higher perceived occupational stress [2]  Lack of control at work was associated with higher perceived occupational stress [2]  Poor perceived rewards at work were associated with higher perceived occupational stress [2]  No significant relationship between team support and stress [2]  No significant relationship between perceived organisational justice and stress [2]  Higher stress in older staff [21]  Higher stress in unmarried staff [21] but another study found no association between marital status and stress, and greater burnout in married staff [8]  Higher stress in less educated staff [21]  No association between gender and stress [8, 26] although females were significantly more likely to report concerns about being injured on the job [26]  No association between rank and stress [8]  No association between sector and stress [8]  No association between area worked in and stress [8]  No association between length of service and stress [26]  No association between being armed (or not) and stress [26]  Smoking more than five cigarettes a day associated with greater stress [21]  High emotional intelligence (i.e. competence in appraising and regulating emotions) associated with less stress [17] |
| Causes of stress | Sleep/fatigue  Managers  Work hours  Other work-related stressors  Danger  Public perceptions  Family-related | Inadequate sleep and rest [17]  Fatigue [26]  Unsupportive managers [8, 17, 20, 26]  Poor leadership including lack of clarity about expectations, and excessive criticisms [8, 17, 24]  Pressure on staff to perform without errors [17]  Leaders perceived to lack time to interact with officers [20]  Work overload [8]  Irregular work hours [8]  Long hours, limited breaks and monotonous work all contributing to fatigue [20]  Not getting leave on a timely basis [17]  Inadequate staffing levels [24, 26]  Poor infrastructure and lack of basic amenities / resources [8, 17, 24]  Low salary / perception of being underpaid [8, 17]  Disadvantageous conditions as compared to police in general [8]  Lack of support from civil authorities [17]  Perceived lack of opportunity for growth or promotion (with work being perceived as hard, monotonous, and carrying little social recognition or reward) [8, 17]  Monotonous tasks including paperwork and bureaucracy [8]  Fear of citizen complaints [8]  Needing to make quick decisions [8]  Tension among different ethnic groups [8]  Rigid, authoritarian workplace culture [8]  Lack of support from colleagues [8]  Difficult physical working conditions [8, 24]  Concerns about being injured [8]  Working with hostile populations in dangerous areas [8, 24]  Negative public perceptions including fear of assaults [8, 24]  Shaming from the public and negative comments about the nature of their work [20]  Felt criticised for being emotionless or uncaring [20] although when they did show emotion they felt they were criticised for being unmasculine [19]  Separation from family, work-family conflict and marital discord [8, 17, 24] |
| Moral challenges | N/A | Performing tasks they did not agree with [8]  Guilt, helplessness, sense of not doing enough to help people [20]  However: no statistical relationship between occupational stress and conflict between personal/occupational values [2] |
| Attitudes towards psychological help | Attitudes towards help-seeking  Attitudes towards providing psychological peer support | Those with more extensive direct trauma exposure exhibited more negative attitudes towards seeking psychological help; almost 40% (of n=168) reported they would feel uneasy if their colleagues knew about their need for psychological support [16]  Whilst peer support could lead to perceived enhancement of compassion, empathy and self-awareness, peer support providers risked developing their own trauma symptoms and felt that providing support was emotionally exhausting and psychologically draining [25] |
| Coping | N/A | Top five coping strategies employed by border police were trying to see the positive in everything; talking with colleagues about problems; using humour; physical activity; and taking part in hobbies [8]  Those who used disengagement coping (strategies aimed at diverting from the stressor and associated emotions, rather than facing them) were more likely to experience stress [26]  Yoga intervention designed to help personnel cope better with job stresses was found to significantly increase vigilance and total duration of sleep, and significantly decrease anxiety and time taken to fall asleep [22, 23] |
